# Supplementary material for: Prolonged Humid Heat Triggers Systemic Inflammation and Stress Signaling: Fluid Intake Modulates NF-κB, p38, JNK2, and STAT3α Pathways
Source: Int J Mol Sci. 2025 May 26;26(11):5114. doi: 10.3390/ijms26115114 (PMC12154057; doi:10.3390/ijms26115114)
Supplement: Supplementary file 1 [file ijms-26-05114-s001.zip › ijms-3645944-supplementary.pdf]

**Supplementary Materials**

# Prolonged Humid Heat Triggers Systemic Inflammation and Stress Signaling: Fluid Intake Modulates NF-κB, p38, JNK2, and STAT3α Pathways

Faming Wang, Caiping Lu, Ying Lei, and Tze-Huan Lei

This document contains 12 pages, 1 figure and 6 tables.

## Supplemental Methods

### Electrolyte concentrations

Plasma electrolyte concentrations ($Na^+$, $K^+$ and $Cl^-$) were measured using an automated ion-selective electrode (ISE) analyzer (BKE-7, Biobase Group, Shandong, China). Calibration and quality proedures were conducted before each batch analysis to ensure precision and accuracy. Results were reported in millimoles per liter (mmol/L). Each sample was measured in triplicate for validation, with intra- and inter-assay coefficients of variation (CVs) maintained below 5%.

### Cardiovascular and body temperature measurements

Heart rate was continuously recorded using a Polar Vantage XL (Polar Electro Oy, Kempele, Finland). Blood pressure was measured using an automated monitor (YE660CR, Yuwell, Yancheng, China) in duplicate at rest, before and after meals, and hourly during exposure. Mean arterial pressure (MAP) was calculated as diastolic blood pressure + 1/3 pulse pressure.

Core temperature was measured with a YSI-401 rectal thermistor (accuracy: 0.1 °C), while mean skin temperature ($T_{sk}$) was recorded at four sites using iButtons (DS1922L, Vadisen Electronic Technology, Shanghai, China). Regional-weighted mean $T_{sk}$ was calculated using Ramanathan's equation (1964).

### Perceptions

Thermal comfort, thermal sensation, wetness perception, thirst sensation, and psychological stress were assessed every 30 minutes throughout the eight-hour extreme heat exposure. Thermal comfort was rated on a seven-point scale ranging from −3 (very uncomfortable) to +3 (very comfortable) (Corgnati *et al.*, 2007). Thermal sensation was recorded on a similar seven-point scale, with −4 representing very, very cold and +4 representing very, very hot (ASHRAE, 2023). Wetness perception was also measured on a seven-point scale, where −3 indicated a very wet sensation and +3 indicated a very dry sensation (Filingeri *et al.*, 2015). Thirst sensation was assessed on a seven-point scale ranging from 1 (not thirsty at all) to 7 (very, very thirsty) (Adams *et al.*, 2020). Psychological stress was evaluated using a ten-point scale, where 0 represented no psychological stress and 10 indicated very severe psychological stress (Williams *et al.*, 2010).

## Supplemental Results

### Cardiovascular responses

Systolic blood pressure, diastolic blood pressure, and mean arterial pressure were all significantly lower at the end of the exposure compared to pre-exposure levels in both the limited fluid intake (LFI)

and full fluid intake (FFI) conditions (all $P<0.01$). However, these measures did not differ significantly between the two fluid intake conditions (all $P>0.70$), and no interaction effects were observed between conditions and time points (all $P>0.10$). In contrast, heart rate was significantly elevated at the end of the exposure compared to pre-exposure ($P<0.01$), but no significant differences were found between the two fluid intake conditions ($P=0.92$), and there was no interaction effect between conditions and time points ($P=0.35$, see Table 1 in the main manuscript).

### Perceptions and psychological stress

Thermal comfort differed significantly between fluid intake conditions and demonstrated a significant interaction effect between conditions and time points ($P=0.014$, see Table 1 in the main manuscript). Participants reported greater comfort in the full fluid intake (FFI) condition than in the limited fluid intake (LFI) condition post-exposure. Thirst sensation also showed significant differences between conditions ($P<0.05$) and across time points ($P=0.02$), with participants reporting lower thirst levels in the FFI condition than in the LFI condition at the end of the exposure. In contrast, thermal sensation did not differ significantly between fluid intake conditions ($P=0.44$) and showed no interaction effect between conditions and time points ($P=0.42$). Psychological stress levels were not significantly different across conditions ($P=0.20$), and no interactional effect was observed between conditions and time points ($P=0.18$).

### Western blot data

**Table S1 Full Western blot membrane images for phosphorylated NF-κB p65 (p-p65), total NF-κB p65, and the loading control (GAPDH) in male (n=6) and female (n=6) participants.** Each set of three lanes represents one participant under Baseline (BL), Limited Fluid Intake (LFI), and Full Fluid Intake (FFI) conditions, respectively, ordered from left to right as follows: M1-BL, M1-LFI, M1-FFI, M2-BL, M2-LFI, M2-FFI, M3-BL, M3-LFI, M3-FFI. GAPDH loading control is shown in corresponding lanes below each NF-κB p65 blot. Images are organized by gender and participant groups: males 1 to 3, males 4 to 6, females 1 to 3, and females 4 to 6.

| p-NF-κB p65 (males 1 to 3) | p-NF-κB p65 (males 4 to 6) |
|---|---|
| 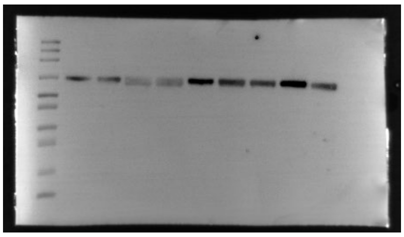 | 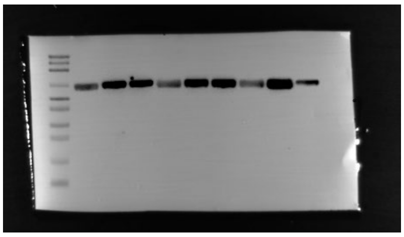 |

| p-NF-κB p65 (females 1 to 3) | p-NF-κB p65 (females 4 to 6) |
|---|---|
| 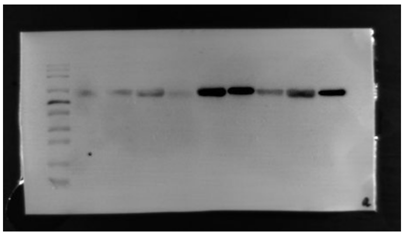 | 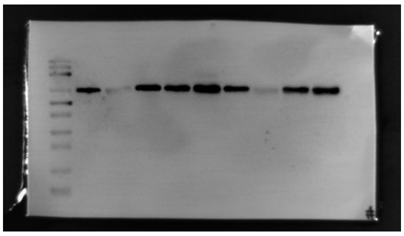 |
| NF-κB p65 (males 1 to 3) | NF-κB p65 (males 4 to 6) |
| 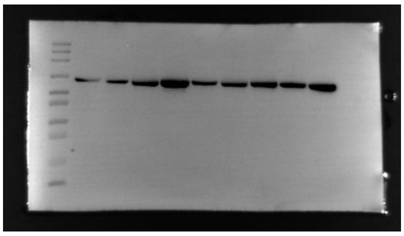 | 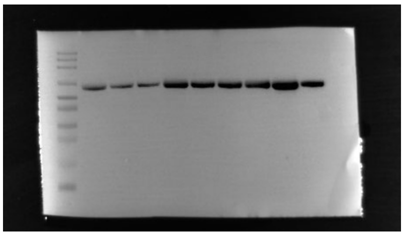 |
| NF-κB p65 (females 1 to 3) | NF-κB p65 (females 4 to 6) |
| 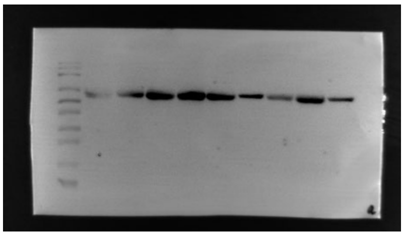 | 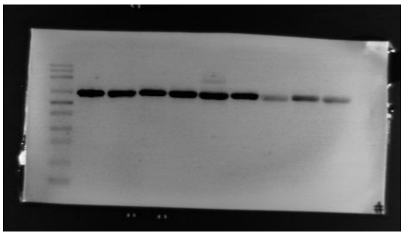 |
| GAPDH (males 1 to 3) | GAPDH (males 4 to 6) |
| 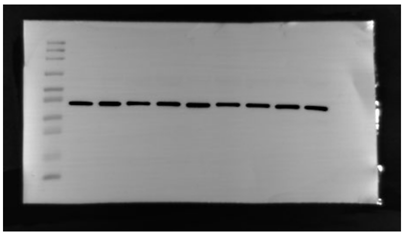 | 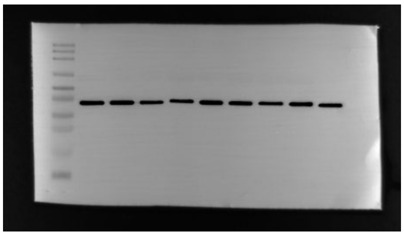 |
| GAPDH (females 1 to 3) | GAPDH (females 4 to 6) |
| 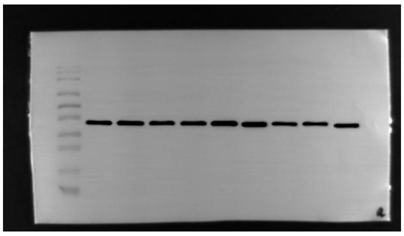 | 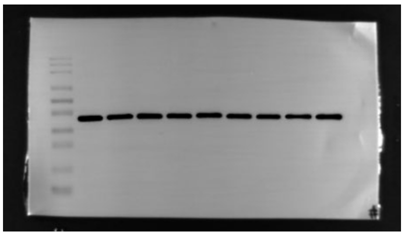 |

Table S2 **Full Western blot membrane images for phosphorylated p38 (p-p38), NF-κB p38, and the loading control (GAPDH) in male (n=6) and female (n=6) participants.** Each set of three lanes represents one participant under Baseline (BL), Limited Fluid Intake (LFI), and Full Fluid Intake (FFI) conditions,

respectively, ordered from left to right as follows: M1-BL, M1-LFI, M1-FFI, M2-BL, M2-LFI, M2-FFI, M3-BL, M3-LFI, M3-FFI. GAPDH loading control is shown in corresponding lanes below each NF-κB p65 blot. Images are organized by gender and participant groups: males 1 to 3, males 4 to 6, females 1 to 3, and females 4 to 6.

| p-p38 (males 1 to 3) | p-p38 (males 4 to 6) |
|---|---|
| 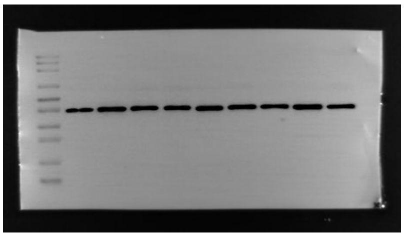 | 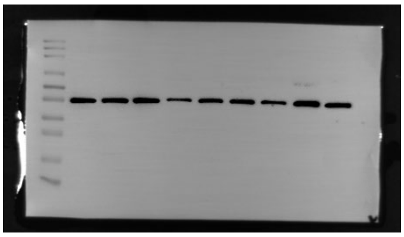 |
| p-p38 (females 1 to 3) | p-p38 (females 4 to 6) |
| 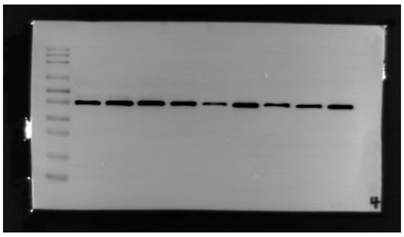 | 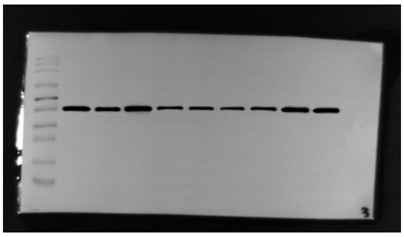 |
| p38 (males 1 to 3) | p38 (males 4 to 6) |
| 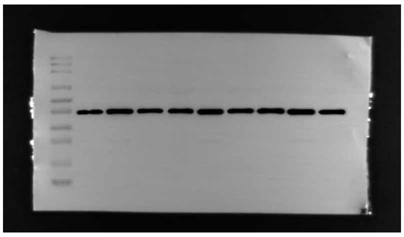 | 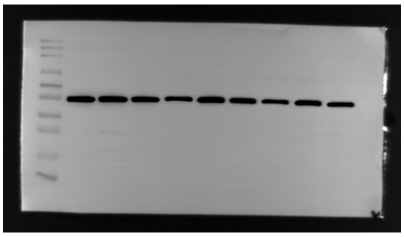 |
| p38 (females 1 to 3) | p38 (females 4 to 6) |
| 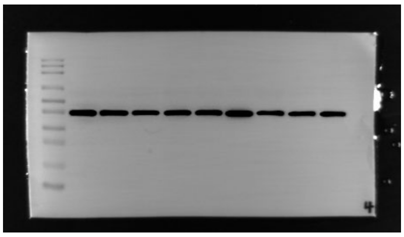 | 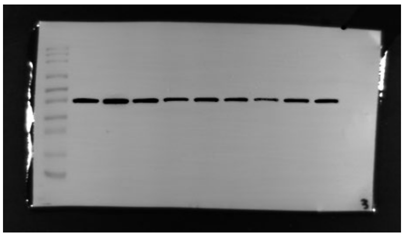 |
| GAPDH (males 1 to 3) | GAPDH (males 4 to 6) |

| | |
|---|---|
| 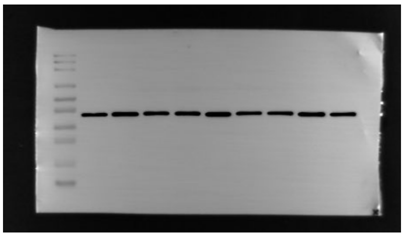 | 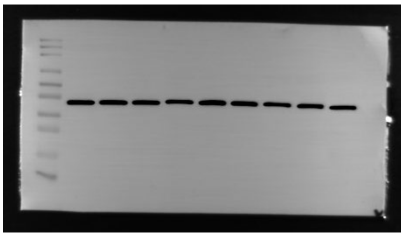 |
| GAPDH (females 1 to 3) | GAPDH (females 4 to 6) |
| 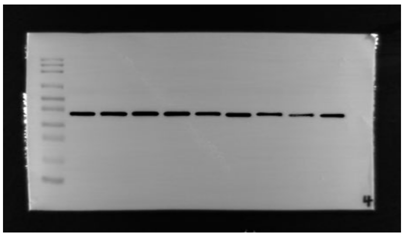 | 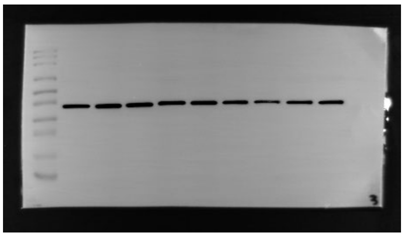 |

**Table S3** **Full Western blot membrane images for phosphorylated JNK (p-JNK), JNK, and the loading control (GAPDH) in male (n=6) and female (n=6) participants.** Each set of three lanes represents one participant under Baseline (BL), Limited Fluid Intake (LFI), and Full Fluid Intake (FFI) conditions, respectively, ordered from left to right as follows: M1-BL, M1-LFI, M1-FFI, M2-BL, M2-LFI, M2-FFI, M3-BL, M3-LFI, M3-FFI. GAPDH loading control is shown in corresponding lanes below each NF-κB p65 blot. Images are organized by gender and participant groups: males 1 to 3, males 4 to 6, females 1 to 3, and females 4 to 6.

| p-JNK (males 1 to 3) | p-JNK (males 4 to 6) |
|---|---|
| 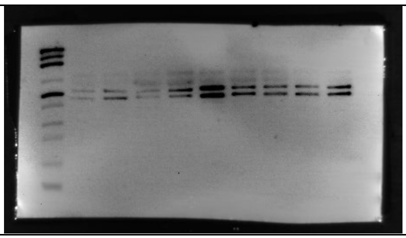 | 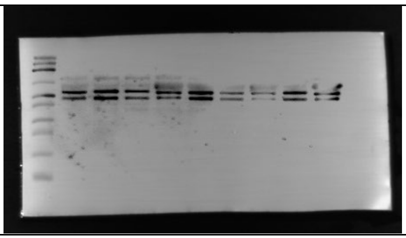 |
| p-JNK (females 1 to 3) | p-JNK (females 4 to 6) |
| 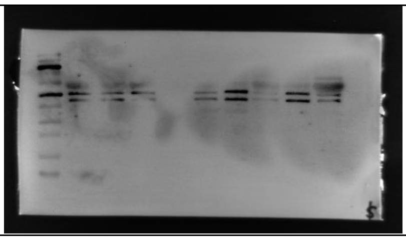 | 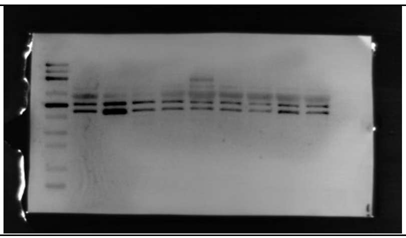 |
| JNK (males 1 to 3) | JNK (males 4 to 6) |

| | |
|---|---|
| 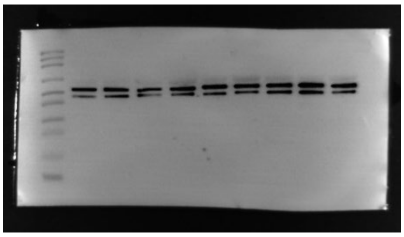 | 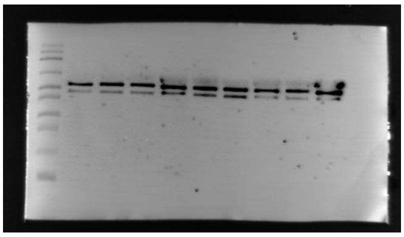 |
| JNK (females 1 to 3) | JNK (females 4 to 6) |
| 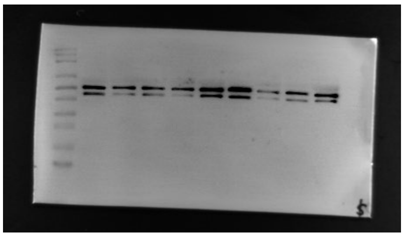 | 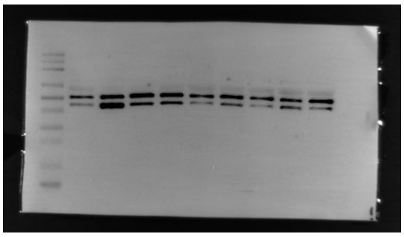 |
| GAPDH (males 1 to 3) | GAPDH (males 4 to 6) |
| 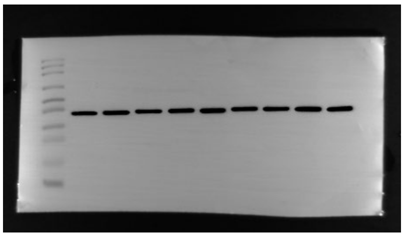 | 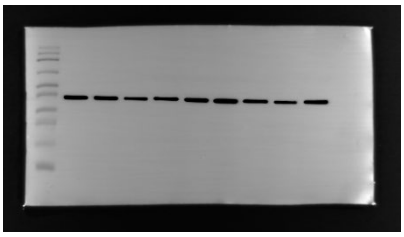 |
| GAPDH (females 1 to 3) | GAPDH (females 4 to 6) |
| 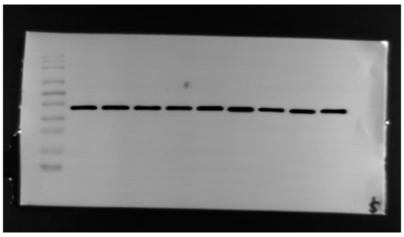 | 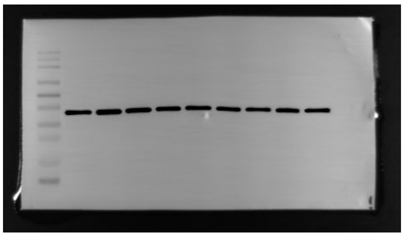 |

Table S4 **Full Western blot membrane images for phosphorylated STAT3 (p-STAT3), STAT3, and the loading control (GAPDH) in male (n=6) and female (n=6) participants.** Each set of three lanes represents one participant under Baseline (BL), Limited Fluid Intake (LFI), and Full Fluid Intake (FFI) conditions, respectively, ordered from left to right as follows: M1-BL, M1-LFI, M1-FFI, M2-BL, M2-LFI, M2-FFI, M3-BL, M3-LFI, M3-FFI. GAPDH loading control is shown in corresponding lanes below each NF-κB p65 blot. Images are organized by gender and participant groups: males 1 to 3, males 4 to 6, females 1 to 3, and females 4 to 6.

| | |
|---|---|
| p-STAT3 (males 1 to 3) | p-STAT3 (males 4 to 6) |

| | |
|---|---|
| 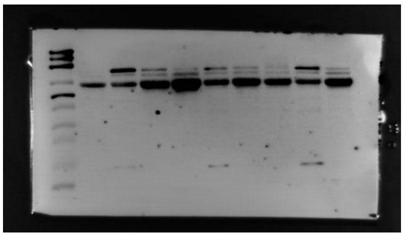 | 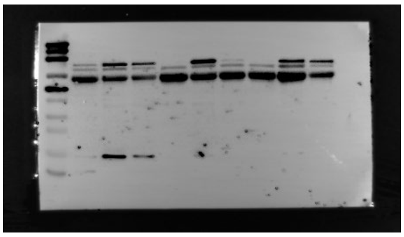 |
| p-STAT3 (females 1 to 3) | p-STAT3 (females 4 to 6) |
| 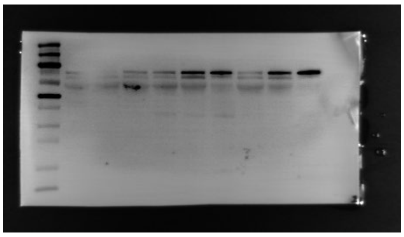 | 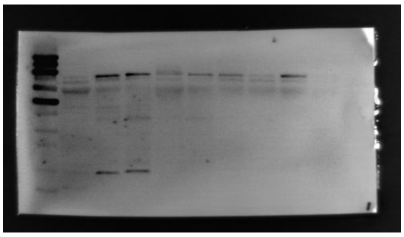 |
| STAT3 (males 1 to 3) | STAT3 (males 4 to 6) |
| 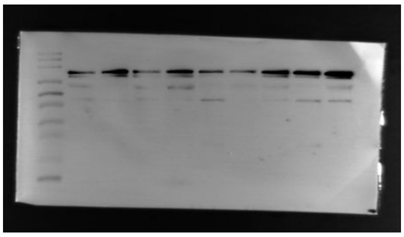 | 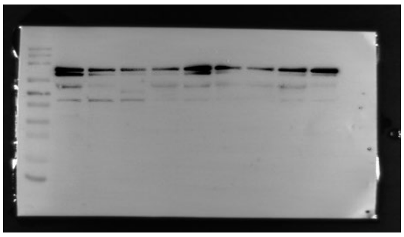 |
| STAT3 (females 1 to 3) | STAT3 (females 4 to 6) |
| 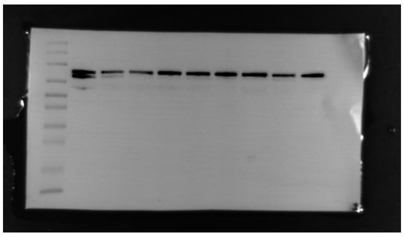 | 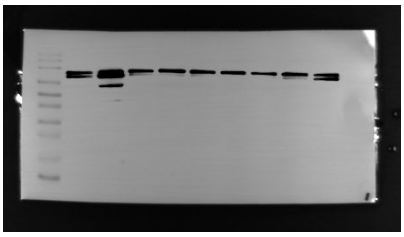 |
| GAPDH (males 1 to 3) | GAPDH (males 4 to 6) |
| 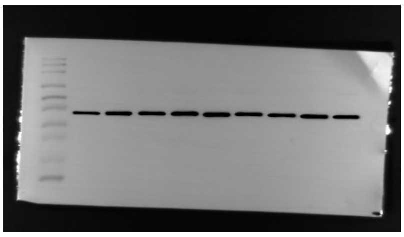 | 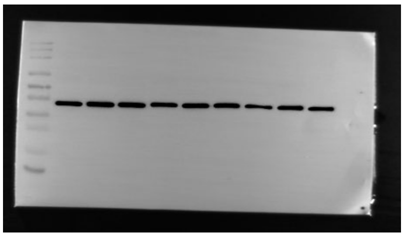 |
| GAPDH (females 1 to 3) | GAPDH (females 4 to 6) |

| | |
|---|---|
| 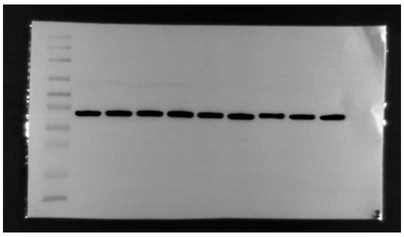 | 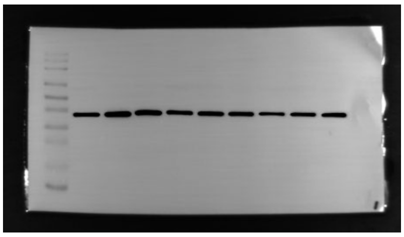 |

**Table S5 Summary of Western blot images showing selected membrane regions for proteins of interest and the loading control (GAPDH) in male (n=6) and female (n=6) participants.**

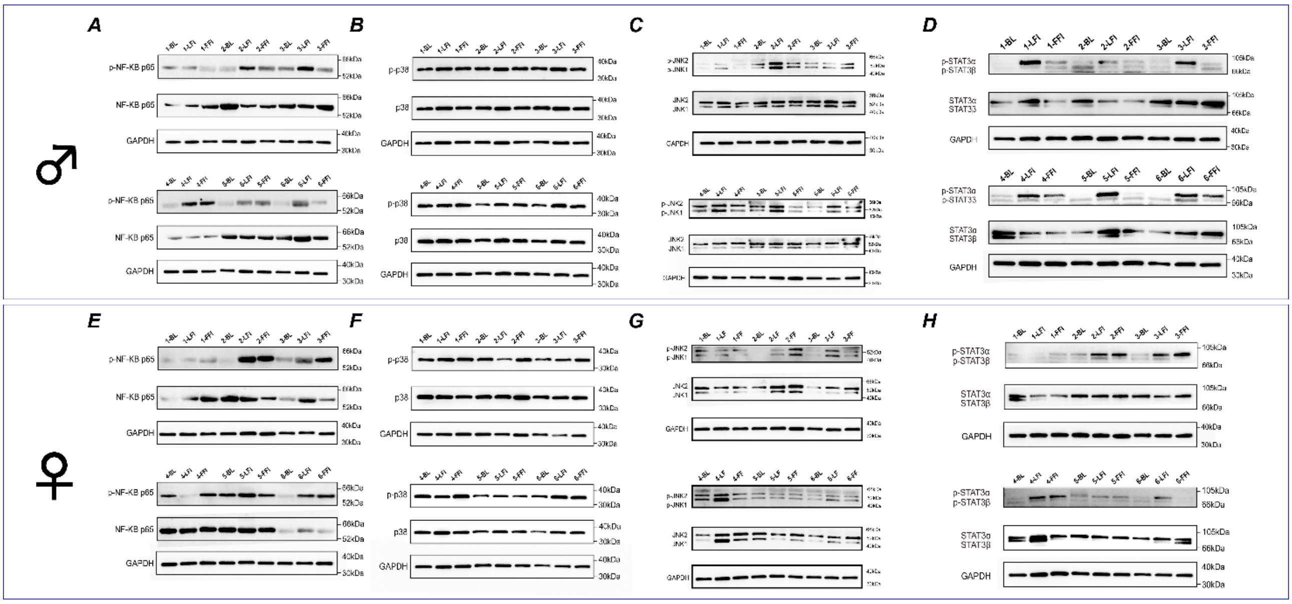

## Sex differences in systemic biomarkers and relative protein expression levels

Statistical comparisons between male and female participants for all systemic biomarkers and relative protein expression levels, both pre- and post-exposure to humid heat, were performed. The results are summarized in Supplementary Table S6. Several significant sex differences were identified. Prior to heat exposure, males showed significantly higher levels of IL-6, ALT, BUN, and MDA ($P \leq 0.009$). Post-exposure, sex differences remained significant for IL-6, IL-1β, IFN-γ, BUN, and MDA ($P \leq 0.001$), with females generally showing greater increases in inflammatory cytokines. In terms of oxidative stress, post-exposure SOD levels were significantly different between sexes ($P = 0.006$). For relative protein expression, post-exposure differences were observed in p-p38/p38 ($P = 0.042$) and p-STAT3α/STAT3α ($P = 0.032$), suggesting potential sex-specific activation in MAPK and JAK-STAT signaling pathways. These findings provide additional insight into sexually dimorphic responses to prolonged heat stress at both systemic and molecular levels.

**Table S6 Statistical analysis (*P*-values) of sex-based differences in systemic biomarkers and relative protein expression levels.**

| | Males vs. Females | |
|---|---|---|
| | Pre-exposure | Post-exposure |
| Systemic biomarker | | |
| Interleukin-6 (IL-6) | $P$=0.001 | $P$<0.001 |
| Interleukin-1β (IL-1β) | $P$=0.407 | $P$<0.001 |
| Interferon-γ (IFN-γ) | $P$=0.336 | $P$<0.001 |
| Tumor necrosis factor-α (TNF-α) | $P$=0.147 | $P$=0.676 |
| Alanine aminotransferase (ALT) | $P$=0.009 | $P$=0.676 |
| Blood urea nitrogen (BUN) | $P$<0.001 | $P$<0.001 |
| Malondialdehyde (MDA) | $P$<0.001 | $P$<0.001 |
| Superoxide dismutase (SOD) | $P$=0.113 | $P$=0.006 |
| Intestinal fatty acid-binding protein (iFABP) | $P$=0.125 | $P$=0.342 |
| Cortisol | $P$=0.330 | $P$=0.920 |
| **Relaive protein expression levels** | | |
| p-NF-κB p65/ NF-κB p65 | $P$=0.627 | $P$=0.668 |
| p-JNK1/ JNK1 | $P$=0.960 | $P$=0.578 |
| p-JNK2/ JNK2 | $P$=0.494 | $P$=0.474 |
| p-p38/ p38 | $P$=0.684 | $P$=0.042 |
| p-STAT3α/ STAT3α | $P$=0.740 | $P$=0.032 |
| p-STAT3β/ STAT3β | $P$=0.071 | $P$=0.365 |
| NF-κB p65/ GAPDH | $P$=0.675 | $P$=0.076 |
| JNK1/ GAPDH | $P$=0.601 | $P$=0.971 |
| JNK2/ GAPDH | $P$=0.977 | $P$=0.165 |
| p38/ GAPDH | $P$=0.157 | $P$=0.249 |
| STAT3α/ GAPDH | $P$=0.171 | $P$=0.217 |
| STAT3β/ GAPDH | $P$=0.742 | $P$=0.671 |

Relative expression of total protein normalized to GAPDH

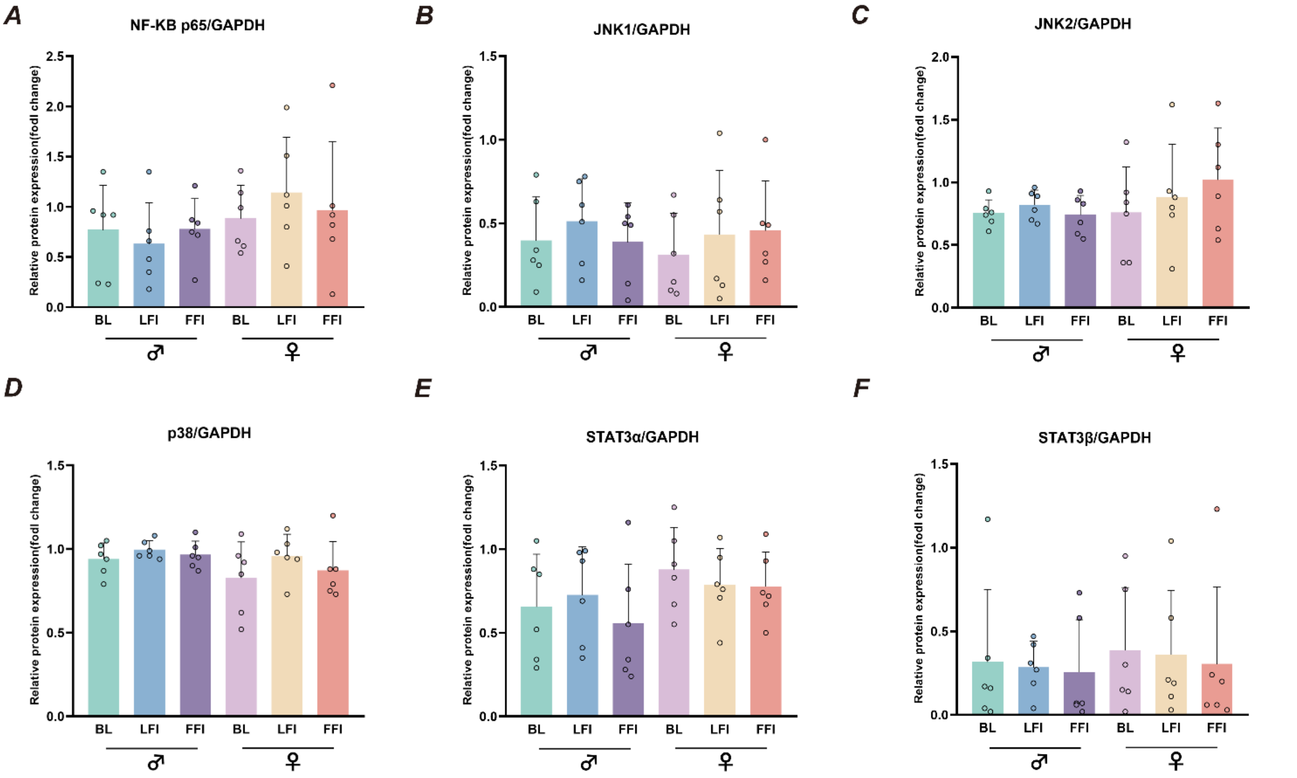


**Figure S1 Relative expression of total NF-κB p65, JNK1, JNK2, p38, and STAT3α/β proteins, normalized to GAPDH as the loading control for accurate quantification, in male (n=6) and female (n=6) participants during eight hours of heat exposure (40 °C, 55% relative humidity) under limited fluid intake (LFI) and full fluid intake (FFI) conditions.** *A*, total NF-κB p65/GAPDH. *B*, total JNK1/GAPDH. *C*, total JNK2/GAPDH. *D*, total p38/GAPDH. *E*, total STAT3α/GAPDH. *F*, total STAT3β/GAPDH. No significant differences were observed in total protein expression across BL, LFI, and FFI conditions (all $P>0.05$).

## Supplemental References

Adams, J. D., Myatich, A. I. & McCullough, A. S. (2020). Thirst as an ingestive behavior: a brief review on physiology and assessment. *Nutr Health* **26**(3), 271-274.

American Society of Heating, Refrigeration and Air Conditioning Engineers (ASHRAE) (2023). Thermal Environmental Conditions for Human Occupancy (ASHRAE Standard 55-2023), ASHRAE, Atlanta, GA.

Filingeri, D., Redortier, B., Hodder, S. & Havenith, G. (2015). Warm temperature stimulus suppresses the perception of skin wetness during initial contact with a wet surface. *Skin Res Technol* **21**(1), 9-14.

Ramanathan, N. L. (1964). A new weighting system for mean surface temperature of the human body. *J Appl Physiol* **19**(3), 531-533.

Williams, V. S., Morlock, R. J. & Feltner, D. (2010). Psychometric evaluation of a visual analog scale for the assessment of anxiety. *Health Qual Life Outcomes* **8**, 57.
